# Very late unusual thrombosis of the remnant pulmonary vasculatures after lung resection complicated by embolic events

**DOI:** 10.1186/s13019-019-1013-9

**Published:** 2019-11-12

**Authors:** Hyun Ju Yoon, Kye Hun Kim, Myung Ho Jeong, Jeong Gwan Cho, Jong Chun Park

**Affiliations:** 1 Department of Cardiovascular Medicine, Chonnam National University Medical School/Hospital, Gwangju, Korea; 2 Director of Echocardiography and Cardiac Imaging Laboratory, Director of heart Failure Clinic Chonnam National University Hospital, 42 Jebong-ro, Dong-gu, Gwangju, 61469 Republic of Korea

**Keywords:** Thrombosis, Vasculature, Lobectomy, Pneumonectomy

## Abstract

**Background:**

Primary thrombosis of the pulmonary vasculatures without extra-pulmonary sources of embolism are uncommon. Here, we report 2 cases of thrombosis of the stump of the remnant pulmonary vasculatures after lung resection complicated by embolic events with review of the literature.

**Case presentation:**

A 75-year-old female was consulted to evaluate cardiac source of embolism for acute cerebral infarction. The patient underwent left upper lobectomy because of lung cancer 2 years ago. Cardiovascular imaging revealed about 1.6 cm × 1.4 cm sized thrombus within the remnant stump of the left superior pulmonary vein. The patient was treated by anticoagulation with warfarin, because the patients refused surgical removal of thrombus. A 57-year-old female who had a history of right pneumonectomy 10 years ago presented with dyspnea. Cardiovascular imaging revealed 1.7 × 1.5 cm sized thrombus in the right pulmonary artery stump and small pulmonary embolism in the left lower segmental pulmonary artery. The patient was treated by long-term anticoagulation with warfarin, and the thrombus and pulmonary embolism were resolved.

**Conclusion:**

The present cases demonstrated that very late thrombosis of the remnant pulmonary vascular structures and subsequent fatal embolic complications can develope even several years later after lung resection. Therefore, the dead space of the remnant vascular structures should be minimized during lung resection surgery, and the developement of delayed thromboembolic complications associated with vascular stump thrombosis should be carefully monitored.

## Background

Pulmonary artery thrombosis (PAT) without extra-pulmonary sources of embolism is uncommon, and pulmonary vein thrombosis (PVT) is rarer than PAT. PVT or PAT has been described in association with tuberculosis, infection, malignancies, and pulmonary fibrosis [1–5]. As the numbers of patients undergoing lung resection increase, there are increasing tendency of case reports regarding the pulmonary vascular stump thrombosis (PVST) [6–10]. PVST usually developed early in the postoperative period [11], but the delayed PVT or PAT after lung resection with embolic complication has rarely been described [12, 13]. Here, we report 2 cases (1 PVT and 1 PAT) of very late pulmonary vascular stump thrombosis after lung resection complicated by embolic events with review of the literature.

## Case presentation

### Case 1

**Fig. 1:**
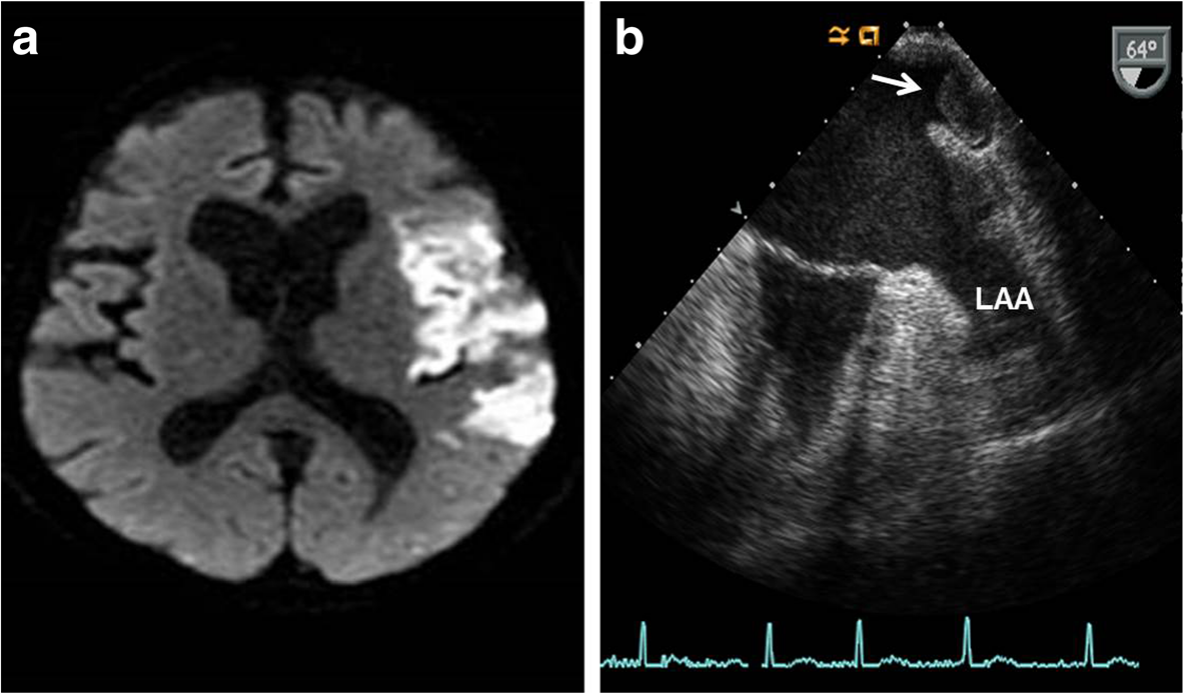
Brain magnetic resonance image demonstrating acute infarction in left middle cerebral aretry territory (**a**). Transesophageal echocardiography demonstrating about 1.6 × 1.4 cm sized thrombus within the remnant stump of the left superior pulmonary vein and no thrombi win left atrial appendage (LAA) (**b**)

**Fig. 2:**
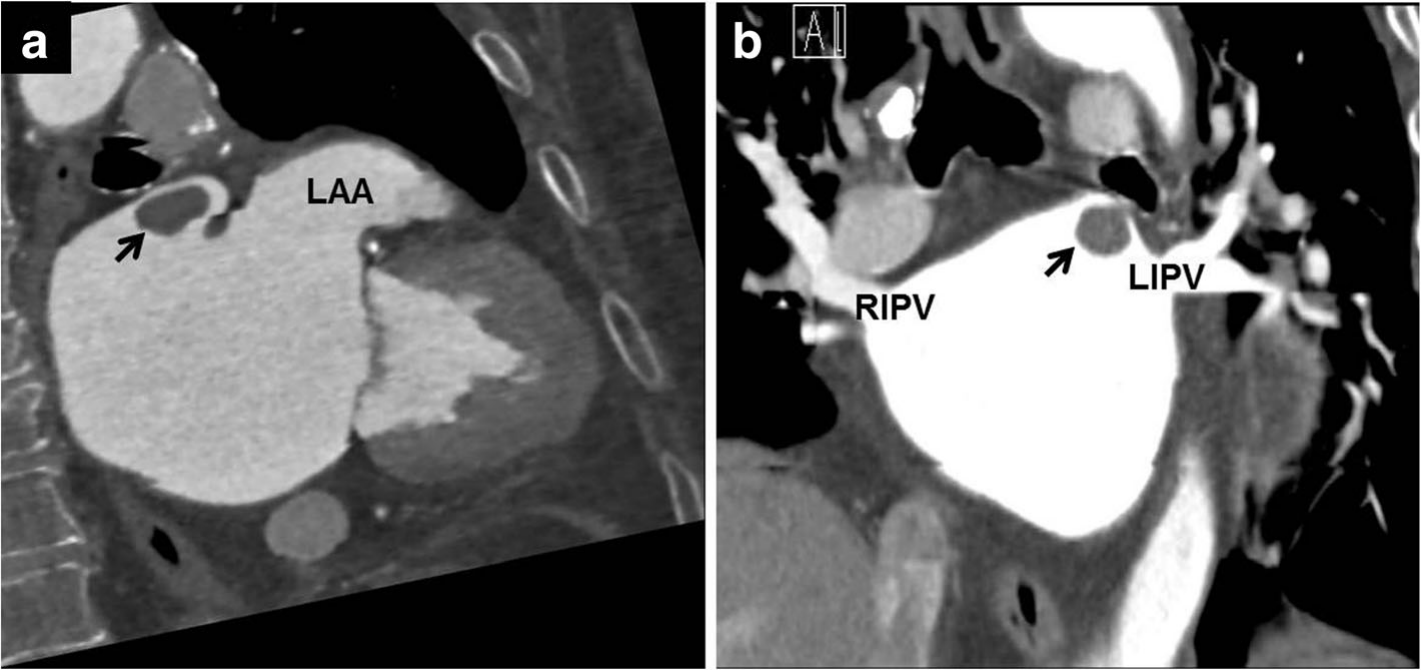
Cardiac computed tomographic angiography demonstrating about 1.6 × 1.4 cm sized thrombus within the remnant stump of the left superior pulmonary vein and no thrombi within left atrial appendage (LAA)(**a**, **b**). RIPV: right inferior pulmonary vein, LIPV: left inferior pulmonary vein

A 75-year-old female was consulted to evaluate cardiac source of embolism for acute cerebral infarction (Fig. 1-a). The patient had a history of left upper lobectomy due to adenocarcinoma of the lung 2 years ago. Lobectomy was performed by thoracotomy through 5th intercostal space. Superior pulmonary vein (PV) was divided and isolated using hand tie. Branches of the pulmonary artery (PA) were divided by ligation and the bronchus was divided. She was a non-smoker and had no other previous history of medical illness. Electrocardiography revealed atrial fibrillation with ventricular rate of 90 beats per minute. Transthoracic echocardiography revealed no visible thrombi within intra-cardiac chambers, but transesophageal echocardiography revealed about 1.6 cm × 1.4 cm sized thrombus within small pouch like structure near the left atrial appendage, but no thrombus in left atrial appendage (Fig. 1-b). Cardiac computed tomographic angiography (CTA) revealed about 1.6 × 1.4 cm sized thrombus within the remnant stump of the left superior pulmonary vein (Fig. 2-a and b). There were no abnormalities in laboratory studies regarding hypercoagulability. To reduce the risk of recurrent embolism, surgical removal of the thrombus was recommended, but the patient refused surgery. The patient was treated by anticoagulation with warfarin.

### Case 2

**Fig. 3:**
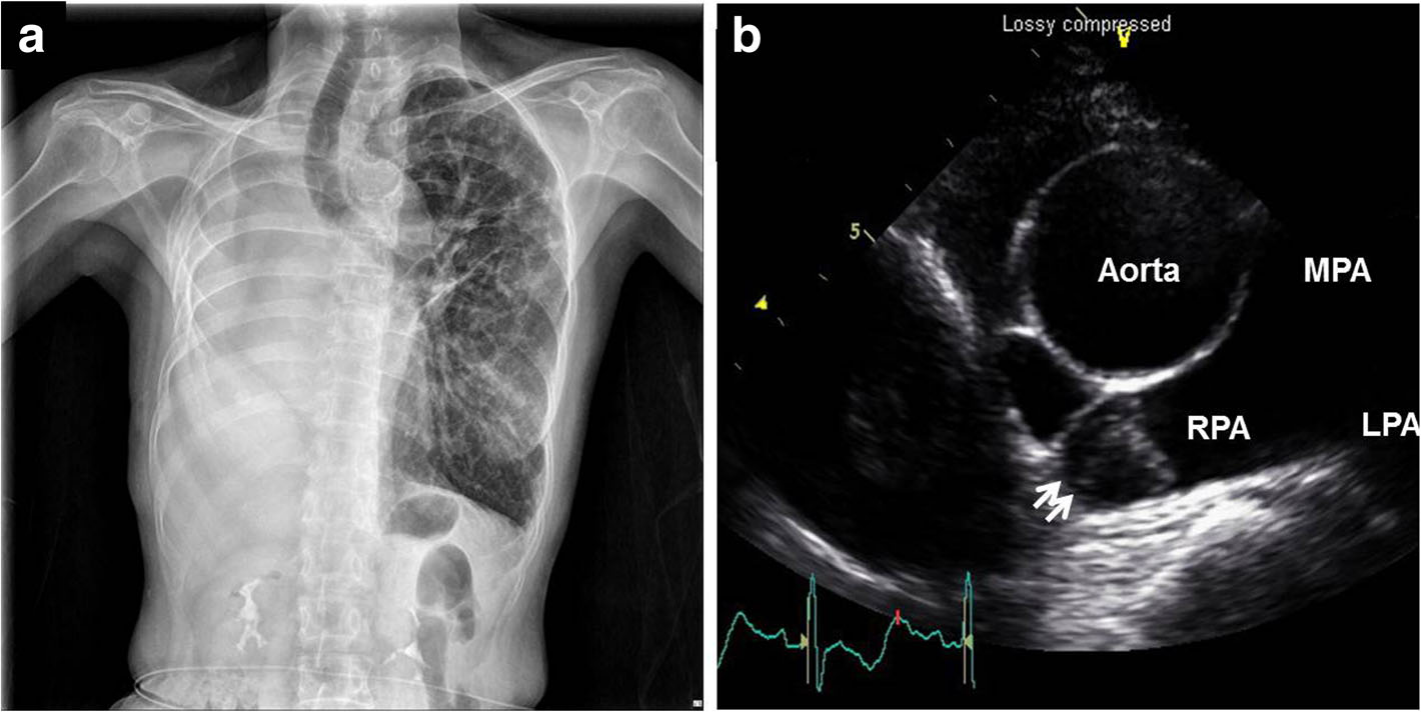
Chest X-ray showing total hazziness on right lung field due to previous pneumonectomy (**a**). Echocardiography demonstrating about 1.7 × 1.5 cm sized round echogenic mass within the dilated stump of the right pulmonary artery (RPA) (**b**). MPA: main pulmonary artery. LPA: left pulmonary aretry

**Fig. 4:**
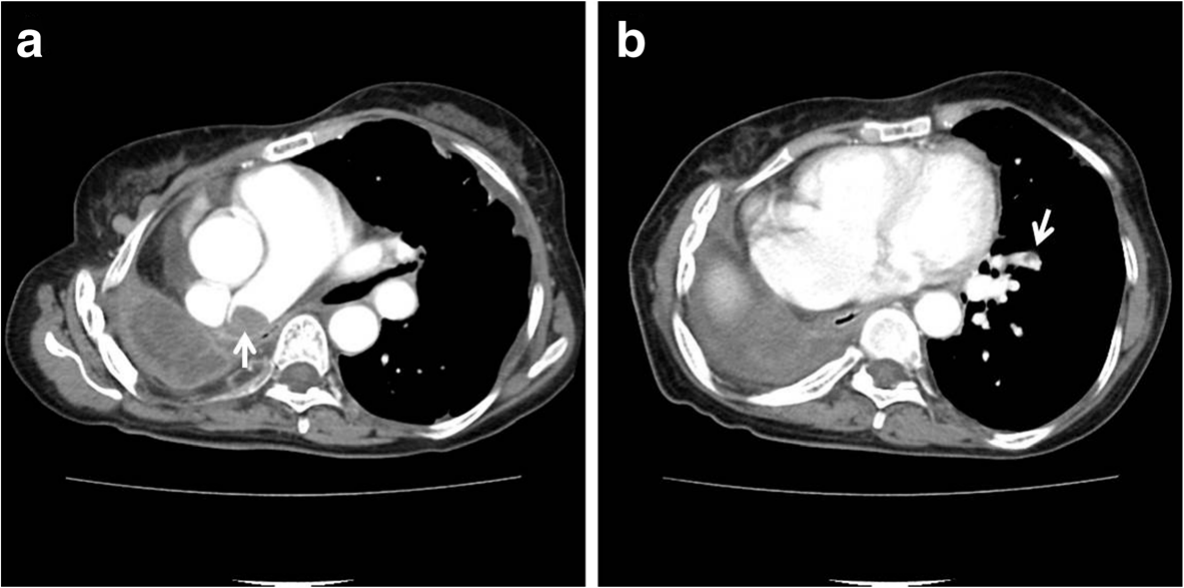
Chest computed tomographic angiography demonstrating about 1.7 × 1.5 cm sized thrombus within the right pulmonary artery stump (**a**) and pulmonary embolism in left lower segmental pulmonary artery (**b**)

**Fig. 5:**
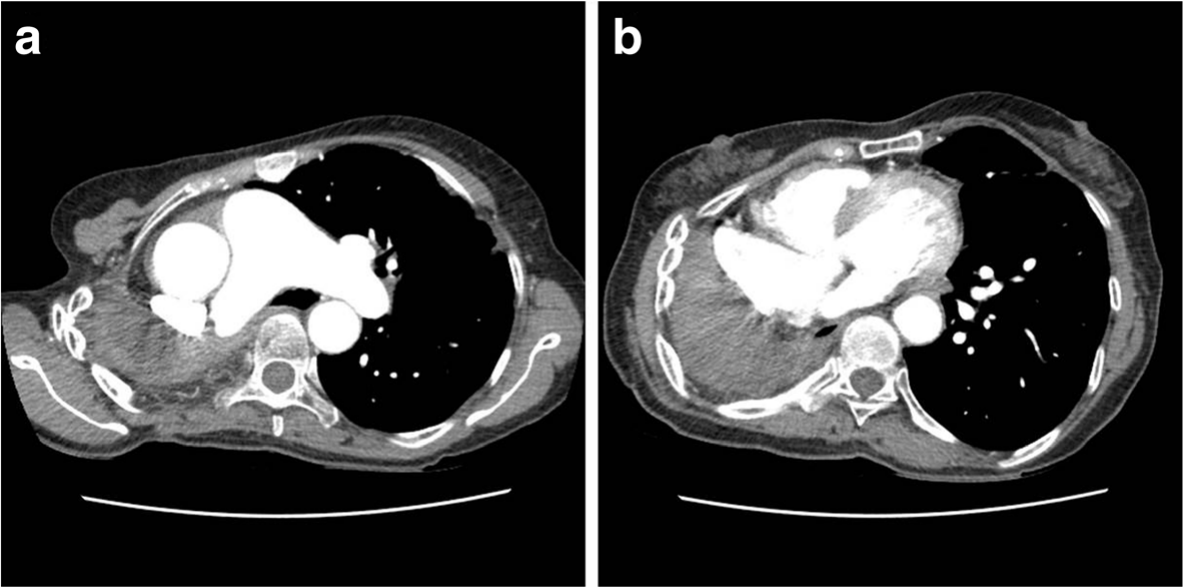
Follow up chest computed tomographic angiography showing the complete resolution of the previously noted thrombus within the right pulmonary aretry stump (**a**) and pulmonary embolism in left lower segmental pulmonary artery (**b**)

A 57-year old female presented with sudden dyspnea. The patient had a history of right pneumonectomy due to chronic empyema and necrotizing pneumonia 10 years ago (Fig. 3-a). Pneumonectomy was performed by posterolateral thoracotomy. Lung’s major vessels were tied off and main bronchus was clamped. Right lung was removed and sutured in remnant vessels. She was a non-smoker and had no other previous history of medical illness except for pneumonectomy. Transthoracic echocardiography showed about 1.7 × 1.5 cm sized round echogenic mass in the dilated stump of right pulmonary artery (Fig. 3-b). Chest CTA revealed about 1.7 × 1.5 cm sized thrombus in the right pulmonary artery stump (Fig. 4-a) and multiple small thrombi in left lower segmental pulmonary artery (Fig. 4-b). There were no abnormalities in laboratory studies regarding hypercoagulability. Follow up chest CTA after 3 months of anticoagulation with warfarin showed completed resolution of the previously noted thrombi in the right pulmonary artery stump and left lower segmental pulmonary artery (Fig. 5a, b).

## Discussion and conclusions

With the widespread and regular use of chest CT after lung resection surgery, unexpected PVST with or without embolic complications has been described sporadically [6–10].

In retrospective CT studies of consecutive patients who underwent lung resection, the incidence of PAT after pneumonectomy was 12% [14], and the incidence of PVT after lobectomy was 3.3% [15]. In a recent study, the incidence of PVST after lung resection for lung cancer was 5.71% (3.85% PAT, 1.85% PVT) [11]. Considering these studies, it is suggested that PVST usually develops early in the postoperative period and PVST is associated with benign clinical course. Actually, in the study of Moon et al. [11], all PVST were incidental findings and none of PVST were associated with any embolic complications. Furthermore, most of PVST showed spontaneous either complete or partial regression, and anticoagulation therapy was needed only a few cases. However, the use of antiocagulation with intravenous heparinization after lung resection in patients with long and large remnant vascular stump seems to be reasonable to prevent PVST, at least in early postoperative period. The use of intermittent pneumatic compression also would be useful in the prevention of PAT, especially in patients who require prolonged immobilization.

In contrary to the results of these studies [11, 14, 15], PVST developed very lately (2 years later in our case 1 and 10 years later in our case 2) from an index lung resection surgery, and both PAT and PVT were associated with embolic complications in the present cases; the cause of dyspnea was contra-lateral pulmonary embolism from right pulmonary artery stump thrombosis in case 2 and the cause of large cerebral infarction was an embolism to the middle cerebral artery from PVT in case 1. In the study of Kalweit et al. [7], pulmonary embolism proved to be a frequent cause of fatality in early post-operative course after lung resection, even though it was unclear whether the source of pulmonary embolism was PVST or not. Also, the delayed PVST after lung resection with embolic complications has been described previously [12, 13], even though very late PVST is rare. Therefore, it is suggested that PVST may develop in any time after an index lung resection surgery, even in several years later, and thus physicians should closely monitor for the possibility of delayed PVST. It is also suggested that PVST is not merely a benign disease; rather it can be associated with embolic complications, sometimes fatal, even though embolic complication of PVST are not common. PAT seems to be more common than in PVT after lung resection surgery, and PAT more commonly developed in post-pneumonectomy patients than in post-lobectomy patients. The reason why the incidence of PAT is higher than PVT is unclear. Larger size of dead space of the remnant PA stump after pneumonectomy than the remnant PV stump after lobectomy would be one of possible explanations. Also, more vascular injury after pneumonectomy than lobectomy would be another possible explanation.

The exact pathophysiologic mechanism of PVST is unclear. Anatomical factors including longer remnant vascular stump, surgical factors including direct vascular injury and vascular distortion, mechanical factors have been suggested as possible precipitating factors [15, 16]. The meticulous surgical technique to minimize the size of the remnant vascular stump and vascular injury would be helpful in preventing the development of PVST. Venous thromboembolism associated with prolonged immobilization after thoracic surgery also could be a possible cause of pulmonary arterial thromboembolism, early ambulation after surgical procedure would be helpful in preventing pulmonary arterial thromboembolism.

Because case 1 presented with cerebral infarction and atrial fibrillation, indefinite lifelong anticoagulation was planned. However, the exact duration of anticoagulation for PVST after lung surgery without identifiable risk factors is not known as in case 2. We planned lifelong anticoagulation with warfarin in case 2 because the anatomic substrate (the dead space of the remnant vascular stump) for thrombosis will be remained and the recurrence of pulmonary embolism may be fatal in the setting of single lung. Further larger studies should be conducted to define the exact duration of anticoagulation in PVST.

In conclusion, the present case demonstrated that very late thrombosis of the remnant pulmonary vascular stump and subsequent fatal embolic complications can develope at any time after lung resection surgery, even several years later. Therefore, the dead space of the remnant vascular structures or vascualr injury should be minimized during lung resection surgery, and the developement of delayed thromboembolic complications associated with PVST should be carefully monitored.

## Data Availability

All information supporting the conclusions of this article is presented in the article.

## Abbreviations

CTA: Computed tomographic angiography
PAT: Pulmonary artery thrombosis
PVT: Pulmonary vein thrombosis

## References

[1] Kim NH, Roldan CA, Shively BK (1993). Pulmonary vein thrombosis. Chest.

[2] Garcia MJ, Rodriguez L, Vandervoort P (1996). Pulmonary vein thrombosis and peripheral embolization. Chest.

[3] Cavaco RA, Kaul S, Chapman T, Casaretti R, Philips B, Rhodes A, Grounds MR (2009). Idiopathic pulmonary fibrosis associated with pulmonary vein thrombosis: a case report. Cases J.

[4] Cha SI, Choi KJ, Shin KM, Lim JK, Yoo SS, Lee J, Lee SY, Kim CH, Park JY (2015). Clinical characteristics of in-situ pulmonary artery thrombosis in Korea. Blood Coagul Fibrinolysis.

[5] Yue M, Zhang X, Zhang H. Ascending aorta thrombosis combined with pulmonary artery thrombosis: a case report. Int J Cardiovasc Imaging. 2019;4 [Epub ahead of print]. 10.1007/s10554-019-01546-4 30830526

[6] Schiller VL, Gray RK (1994). Causes of clot in the pulmonary artery after pneumonectomy. Am J Roentgenol.

[7] Kalweit G, Huwer H, Volkmer I, Petzold T, Gams E (1996). Pulmonary embolism: a frequent cause of acute fatality after lung resection. Eur J Cardiothorac Surg.

[8] Kotoulas C, Lachanis S (2009). Embolism of the pulmonary artery stump after right pneumonectomy. Interact Cardiovasc Thorac Surg.

[9] Barbetakis N, Asteriou C, Kleontas A (2011). Post-lobectomy pulmonary artery stump thrombosis: how dangerous is it?. Ann Thorac Surg.

[10] Sato W, Watanabe H, Sato T, Iino K, Sato K, Ito H (2014). Contralateral pulmonary embolism caused by pulmonary artery stump thrombosis after pneumonectomy. Ann Thorac Surg.

[11] Moon MH, Beck KS, Moon YK, Park JK, Sung SW (2017). Incidence and clinical features of the incidentally found vascular stump thrombus during routine follow up after oncologic lung surgery. PLoS One.

[12] Thomas PA, Doddoli C, Barlesi F, Giudicelli R, Fuentes P (2006). Late pulmonary artery stump thrombosis with post embolic pulmonary hypertension after pneumonectomy. Thorax.

[13] Sawalha L, Mador MJ (2015). Delayed post-lobectomy pulmonary artery stump thrombosis. Respir Med Case Rep.

[14] Kim SY, Seo JB, Chae EJ, Do KH, Lee JS, Song JW, Song KS, Lim TH (2005). Filling defect in a pulmonary arterial stump on CT after pneumonectomy: radiologic and clinical significance. AJR Am J Roentgenol.

[15] Ohtaka K, Hida Y, Kaga K, Takahashi Y, Kawase H, Hayama S, Ichimura T, Senmaru N, Honma N, Matsui Y (2014). Left upper lobectomy can be a risk factor for thrombosis in the pulmonary vein stump. J Cardiothorac Surg.

[16] Mumoli N, Cei M (2012). Idiopathic pulmonary vein thrombosis. J Emerg Med.

